# Development and validation of the Brazilian version of the Attitudes to Aging Questionnaire (AAQ): An example of merging classical psychometric theory and the Rasch measurement model

**DOI:** 10.1186/1477-7525-6-5

**Published:** 2008-01-21

**Authors:** Eduardo Chachamovich, Marcelo P Fleck, Clarissa M Trentini, Ken Laidlaw, Mick J Power

**Affiliations:** 1 Post-Graduate Program of Psychiatry, Universidade Federal do Rio Grande do Sul, Brazil; 2 Section of Clinical and Health Psychology, University of Edinburgh, UK

## Abstract

**Background:**

Aging has determined a demographic shift in the world, which is considered a major societal achievement, and a challenge. Aging is primarily a subjective experience, shaped by factors such as gender and culture. There is a lack of instruments to assess attitudes to aging adequately. In addition, there is no instrument developed or validated in developing region contexts, so that the particularities of ageing in these areas are not included in the measures available. This paper aims to develop and validate a reliable attitude to aging instrument by combining classical psychometric approach and Rasch analysis.

**Methods:**

Pilot study and field trial are described in details. Statistical analysis included classic psychometric theory (EFA and CFA) and Rasch measurement model. The latter was applied to examine unidimensionality, response scale and item fit.

**Results:**

Sample was composed of 424 Brazilian old adults, which was compared to an international sample (n = 5238). The final instrument shows excellent psychometric performance (discriminant validity, confirmatory factor analysis and Rasch fit statistics). Rasch analysis indicated that modifications in the response scale and item deletions improved the initial solution derived from the classic approach.

**Conclusion:**

The combination of classic and modern psychometric theories in a complementary way is fruitful for development and validation of instruments. The construction of a reliable Brazilian Attitudes to Aging Questionnaire is important for assessing cultural specificities of aging in a transcultural perspective and can be applied in international cross-cultural investigations running less risk of cultural bias.

## Background

The world is experiencing a profound and irreversible demographic shift as older people are living longer and healthier than ever before [1,2]. The world's older adult population is estimated to show a threefold increase over the next fifty years, from 606 million people today to 2 billion in 2050 [2]. In 2002, older people constituted 7 per cent of the world's population and this figure is expected to rise to 17 per cent globally by 2050 [3]. The most dramatic increases in proportions of older people are evident in the oldest old section of society (people aged 80 years plus) with an almost fivefold increase from 69 million in 2000 to 377 million in 2050 [4].

The World Health Organisation has described this demographic shift as a major societal achievement, and a challenge [5]. The increase in longevity is being experienced in the developed and the developing world alike, but where the developed world grew rich before it grew old, the developing world is growing old before it has grown rich [5]. While older people are living longer they are generally remaining healthier with an increase in percentage of life lived with good health. Nonetheless older people are still seen as net burdens on society rather than net contributors to it [5,6].

Quantifying the raise of proportion of old adults in the world population is relevant but insufficient. It is also important to study the quality of this increase. The experience of ageing is primarily subjective and depends on several factors, such as gender, physical condition, environment, behavioural and social determinants, psychological strategies and culture [5,7-10]. Culture is considered particularly relevant since it shapes the way in which one ages due to the influence it has on how the elderly are seen by a determined context [5]. Moreover, the cultural aspects could be understood as a pathway through which the external aspects would impact on ageing experiences.

Authors state that the vast majority of research and discussion is done by young adults, whereas older adults would be the most indicated to propose adequate ways of doing it [11,12]. Bowling and Diepe argue that lay viewers are important for testing the validity of existing models and measures, since most of the discussion tends to reflect only the academic point of view [13]. Even though investigating the ageing process has been a topic of increased interest, there is a remarkable lack of well-designed and tested instruments to assess it. The few developed so far are either not specific to cover older adult's experiences or have been exclusively carried out in developed countries [14]. As far as we are aware, there is no instrument developed or validated in developing region contexts, so that the particularities of ageing in these areas are not included in the measures available.

To address this issue, the WHOQOL Group has developed the AAQ instrument under a simultaneous methodology [15], which ensured the participation of different centres throughout the world (described in details in Laidlaw *et al*, 2007) [14]. Briefly, the development process included centres from distinct cultural contexts in qualitative item generation, piloting and field testing. The applied methodology followed the one established by the World Health Organization Quality of Life Group [16,17] for the development and adaptation of quality of life measures and was used for the development of the WHOQOL-OLD module [18,19].

Regarding development of new measures or validation of existing ones, new approaches have been added to the traditional ones in order to expand the scale's properties beyond reliability and validity [20]. The Rasch model has been adopted since it permits that data collected may be compared to an expected model and allows testing other important scale features, such as reversed response thresholds and differential item functioning.

The present paper aims to illustrate the potential combination of classical psychometric theory and Rasch Analysis in the validation of the AAQ instrument in a Brazilian sample of older adults.

## Methods

### Pilot study

The pilot study followed the methodology applied by the WHOQOL Group in developing quality of life measures [16,17]. This includes translation and back-translation of the items and instructions by distinct professionals, as well as semantic and formal examination by the coordinator centre. Convenience sampling was used. The main purpose of this stage was to collect data about the item performance in order to produce a reduced version after refinement. The combination of classical and modern (item response theory) statistical analyses was used at this point. A set of 44 items were tested in an opportunistic sample of 143 subjects (age range 60–99, 59% female, 55% living alone, and 59% considered themselves subjectively healthy). Patients with dementia, other significant cognitive impairments and/or terminal illness were excluded. Data collected at this stage were sent to the coordinator centre to be merged with other centres' information.

Statistical analyses were carried out to check the items regarding missing values, item response frequency distributions, item and subscale correlations and internal reliability. No missing values were found in any of the 44 items in the Brazilian sample. The analysis of the pooled international data indicated the need of item refinement, which resulted in a 38-item version to be tested in the field trial (see Laidlaw *et al *(2007) for more details on this refinement stage) [14].

### Field trial

The Brazilian Field Trial was carried out with a non-probabilistic opportunistic sample of 424 older adults recruited from a university hospital, community houses and nursing homes, elderly community groups, and their own homes. Subjects were invited to take part of the study and were asked to indicate other potential participants (snowball strategy). Sampling was used according to previous stratification determined by subjective perception of health status (50% healthy ones and 50% unhealthy ones), gender (50% female) and age (60–69 years of age, 70–79 years of age and over 79 years of age). Subjective perception of health status was assessed by the question "In general, you consider yourself healthy or unhealthy?", regardless of the objective health condition. Exclusion criteria followed the ones used in the pilot study [14]. The purpose of stratification was to ensure a minimal representation in each subgroup to make further analyses possible.

This version comprised the 33 items from the Pilot Study plus 5 items added by the Coordinator Centre (Edinburgh) in order to cover areas not sufficiently investigated by the original format. These 5 items were translated and back-translated and re-examined by the coordinator centre. In addition, subjects completed a socio-demographic form and the Geriatric Depression Scale 15-item version [21].

### Statistical analysis

The combination of classical and modern psychometric approaches was applied. The descriptive data analysis was used to determine item response frequency distributions, missing values analysis, item and subscales correlations and internal reliability analyses. Exploratory and Confirmatory Factor analysis were performed to assess whether the Brazilian data fit the international pooled model. Finally, an IRT approach, in particular, that of the Rasch model as implemented in the RUMM 2020 program [22], was used to examine the performance of items in the Brazilian dataset.

## Results

### Demographics

Table 1 describes the socio-demographic characteristics of both the Brazilian and the international samples. Note that the international sample is composed of the data collected in all centers apart from Brazil. Chi-Square and Independent T-tests were carried out to check statistical differences across both samples. Following the detection of differences in gender and educational level distributions, as well as in the mean depression level, an Independent T-test was then run to compare means of the three original AAQ factor scores (as described in Laidlaw *et al*, 2007) [14] between the two samples. Briefly, the factor scores were calculated by summing the items included in each factor. Results indicate statistical differences in all three factor scores, as well as in the overall score.

**Table 1:** Socio-demographic characteristics of Brazilian and International Samples

	**Brazilian sample n = 424**	**International sample n = 5238**	**P**
	**N (%) or M (SD)**	**N (%) or M (SD)**	
**Age**			**0.640**^**a**^
60–69 years old	173 (40.9)	1983 (39.1)	
70–-79 years old	153 (36.2)	1948 (38.4)	
80 or + years old	97 (22.9)	1141 (22.5)	
**Gender**			**0.013**^**b**^
Male	152 (35.8)	2191 (42.1)	
Female	272 (64.2)	3014 (57.9)	
**Perceived Health Status**			**0.215**^**b**^
Healthy	286 (67.5)	3573 (70.8)	
Unhealthy	138 (32.5)	1476 (29.2)	
**Marital Status**			**0.275**^**a**^
Single	29 (6.8)	275 (5.5)	
Married	212 (50.0)	2688 (54)	
Separated	30 (7.1)	397 (8)	
Widowed	128 (30.2)	1371 (27.5)	
**Educational Level**			**0.000**^**a**^
Illiterated	7 (1.7)	138 (2.7)	
Basic Level	165 (38.9)	1441 (28.3)	
High School	110 (25.9)	1956 (38.4)	
College	90 (21.2)	1449 (28.5)	
**Depression Level**			**0.041**^b^
GDS 15	3.99 (2.91)	3.68 (2.69)	

^a ^Chi-Square test; ^b ^independent t test

An Ancova analysis was then carried out to assess the extent to which the interaction among depression, gender and educational level was implied in determining differences in the scores (overall and each factor). Comparisons between both samples were run to rule out the possibility that differences in posterior factor analyses are due to distinct sample characteristics. Table 2 illustrates the Ancova findings, indicating that the statistical difference in the distribution of these variables between the two samples does not interfere significantly with the score variations [23].

**Table 2:** Ancova analyses including Educational level, gender and depression between Brazilian and International Samples

**Interaction**	**Means Br**	**Means Int**	**F**	**P**	**Partial Eta Sq.**
**Total score**					
Gender (m/f)	132.8/137.3	129.9/128.9	1.231	.267	.000
Ed Level (high/low)	139.3/134.5	132.1/128.3	18.96	.000	.004
Depression (≤5/>5)	141.2/119.4	134.4/110.8	2914.5	.000	.430
Gender × Ed Level × Depression	-	-	.084	.773	.000
**Factor I score**					
Gender	49.4/51.1	49.7/48.5	13.5	.000	.003
Ed Level	51.8/50.5	50.7/48.4	37.3	.000	.007
Depression	53.1/42.8	51.4/40.0	2233.7	.000	.352
Gender × Ed Level × Depression	-	-	.001	.971	.000
**Factor II score**					
Gender	50.3/52.7	49.9/49.8	.073	.787	.000
Ed Level	54.1/51.1	51.2/49.4	14.59	.000	.003
Depression	54.0/45.3	51.9/42.3	1746.4	.000	.301
Gender × Ed Level × Depression	-	-	1.25	.263	.000
**Factor III score**					
Gender	33.0/33.4	30.2/30.3	1.80	.179	.000
Ed Level	33.3/33.3	30.2/30.9	2.29	.130	.000
Depression	34.0/31.2	31.0/28.7	304.9	.000	.067
Gender × Ed Level × Depression	-	-	.321	.571	.000

### Descriptives

Summary descriptives statistics for item analyses are shown in Table 3. There is low frequency of missing values across the items. Comparison of the missing frequencies with the international dataset showed a lower frequency in the Brazilian sample.

**Table 3:** Descriptive analysis of the set of 38 items in the Brazilian sample (n = 424)

**Item content**	**Mean**	**SD**	**MV(%)**	**Distribution**					**Skew**	**Kurt**
						
				**1**	**2**	**3**	**4**	**5**		
1 People as old as they feel	3.42	1.18	0	7.3	19.3	13.7	42.9	16.7	-.52	-.76
**2 Better able to cope with life**	**3.81**	**.781**	**0**	**.9**	**6.4**	**16.7**	**62.3**	**16.7**	**.781**	**1.411**
3 Old age time of illness	2.24	1.015	0	25	42.2	17.5	14.4	.9	.554	-.549
**4 Privilege to grow old**	**3.96**	**.93**	**0**	**1.9**	**6.6**	**14.6**	**47.6**	**29.2**	**-.96**	**.82**
5 Interested in new technology	3.0	1.02	0	6.8	27.1	30.7	30.2	5.2	-.087	-.748
6 Interested in love	3.64	.881	0	2.4	8	25.2	52.4	12	-.766	.666
**7 Old age is a time of loneliness**	**2.27**	**1.029**	**0**	**23.3**	**44.1**	**16.3**	**14.6**	**1.7**	**1.029**	**-.409**
**8 Wisdom comes with age**	**3.76**	**.872**	**0**	**1.4**	**8.7**	**18.2**	**55.9**	**15.8**	**.872**	**.664**
**9 Pleasant things about growing older**	**3.79**	**.826**	**0**	**1.2**	**7.8**	**16.5**	**60.1**	**14.4**	**.826**	**1.082**
**10 Old age depressing time of life**	**2.38**	**.997**	**0**	**19.1**	**41.5**	**22.2**	**16.5**	**.7**	**.997**	**-.752**
11 Capacities and abilities decline with age	3.54	.870	.2	3.1	11.6	18.4	62.4	4.5	-1.145	.832
**12 Important to take exercise at any age**	**4.26**	**.666**	**0**	**.7**	**1.4**	**4**	**59**	**34.9**	**.666**	**4.101**
**13 Growing older easier than I thought**	**3.41**	**.981**	**0**	**5.9**	**9.7**	**30.2**	**45.8**	**8.5**	**.981**	**.261**
**14 More difficult to talk about feelings**	**2.44**	**1.118**	**0**	**25.9**	**26.4**	**26.9**	**19.1**	**1.7**	**1.118**	**-1.073**
**15 More accepting of myself**	**3.10**	**1.097**	**0**	**10.1**	**18.4**	**29.2**	**35.6**	**6.6**	**1.097**	**-.674**
**16 I don't feel old**	**3.40**	**1.132**	**0**	**8.3**	**12.3**	**25.2**	**39.4**	**14.9**	**1.132**	**-.389**
**17 Old age mainly as a time of loss**	**2.17**	**1.137**	**0**	**38.4**	**23.3**	**22.2**	**14.6**	**1.4**	**1.137**	**-.970**
18 Personal beliefs mean more as I grow older	3.61	1.18	0	9.5	8.5	16	44.8	21.5	-.868	-.051
**19 My identity is not defined by my age**	**3.29**	**1.133**	**.2**	**11.6**	**9.9**	**25**	**44.3**	**9**	**1.133**	**-.333**
**20 More energy than I expected for my age**	**3.32**	**1.063**	**.2**	**6.9**	**16.1**	**23.3**	**44.7**	**8.7**	**1.063**	**-.408**
**21 Loss physical independence as I get older**	**2.80**	**1.156**	**0**	**18.2**	**20.3**	**28.5**	**29**	**3.8**	**1.156**	**-1.039**
**22 Physical health problems don't hold me back**	**3.25**	**1.176**	**.2**	**11.1**	**15.1**	**22.2**	**40.4**	**11.1**	**1.176**	**-.686**
23 Unhappy with changes in physical appearance	2.16	1.128	.2	38.5	23.9	21.7	14.7	1.2	.496	-.979
**24 More difficult to make new friends**	**2.08**	**1.162**	**0**	**44.8**	**19.6**	**18.6**	**15.8**	**.9**	**1.162**	**-1.030**
**25 Pass on benefits of experience**	**3.94**	**.821**	**.5**	**1.4**	**4.3**	**15.4**	**56.6**	**22.3**	**.821**	**1.618**
26 Fear loosing financial independence	2.36	1.287	.2	38.1	17	19.9	21	4	-.358	-1.239
27 Time to do things that really interest me	3.43	1.00	.5	5.9	11.1	26.1	47.7	9.5	-.741	.109
28 Want continue doing work long as possible	3.58	1.23	.2	10.2	9.5	16.8	39.2	24.3	-.760	-.372
29 Worried I'll become a financial burden to family	2.23	1.28	.2	40.9	21.5	16.5	15.6	5.4	.636	-.855
**30 Believe my life has made a difference**	**3.73**	**.847**	**.2**	**2.4**	**5.4**	**22.2**	**56.5**	**13.5**	**.847**	**1.369**
31 Just as meaning now as always	3.73	.931	.5	2.4	9.7	16.8	54.5	16.6	-.882	.602
**32 Don't feel involved in society**	**2.55**	**1.184**	**.5**	**25.9**	**21.5**	**25.5**	**24.3**	**2.4**	**1.184**	**-1.229**
**33 Want to give a good example**	**4.07**	**.735**	**.2**	**1.4**	**1.9**	**9.7**	**62.6**	**24.3**	**.735**	**3.619**
**34 I feel excluded because of my age**	**2.17**	**1.143**	**.2**	**39.2**	**20.8**	**25**	**13**	**1.9**	**1.143**	**-.928**
35 Future fills me with dread	2.12	1.15	.5	41	23	21.8	11.1	3.1	.673	-.597
**36 Health is better than expected for my age**	**3.38**	**1.122**	**.2**	**8.7**	**13**	**22**	**44.4**	**11.8**	**1.122**	**-.361**
**37 Keep myself fit and active by exercising**	**3.02**	**1.284**	**.5**	**17.1**	**17.8**	**23.7**	**29.1**	**12.3**	**1.284**	**-1.077**
38 Important relationships become more satisfying	3.26	1.03	.2	7.8	12.3	34.5	36.9	8.5	-.499	-.195

Items in bold were retained in the international final version

### Exploratory Factor Analysis

Data were initially examined through Exploratory Factor Analysis (Principal Component Analysis with Varimax Rotation). Extraction strategy included selecting factors with eigenvalues higher than 1 (and confronted to Monte Carlo Parallel Analysis to control for spurious findings) and scree plot observation [24-26]. The three-factor solution (indicated both by the Kaiser Rule plus Parallel Analysis and Scree Plot) accounted for 34.45% of the total variance, whereas in the international sample the same structure was responsible for 32.74%.

**Figure 1:**
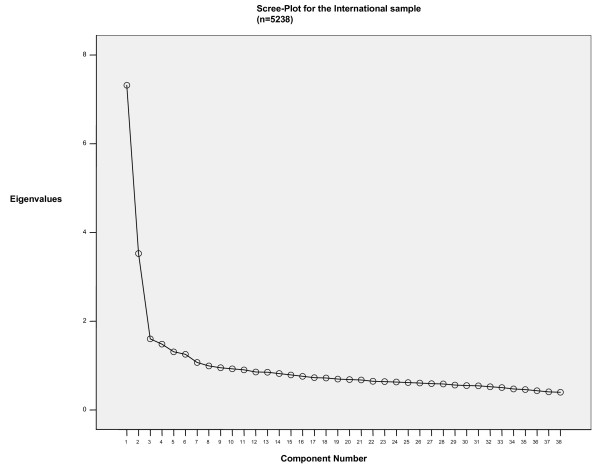
Scree-Plot for the International Sample (n = 5238).

**Figure 2:**
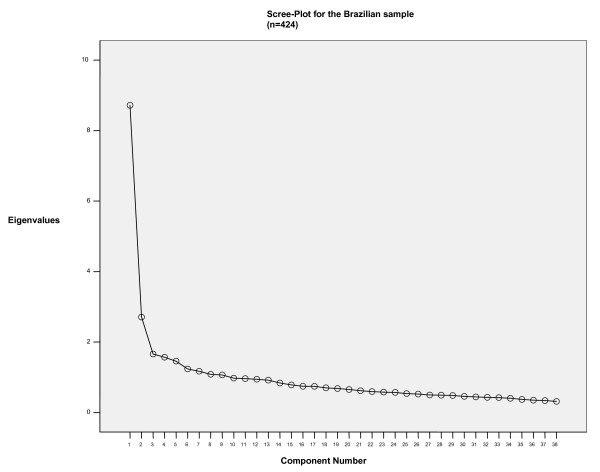
Scree-Plot for the Brazilian sample (n = 424).

Figures 1 and 2 show the Scree Plot for both the Brazilian and International Samples, indicating remarkable similarities between both.

EFA findings were compared to the international ones. There is a great similarity of the item loadings when comparing to the EFA run in the international dataset. Out of 38 items, only five (items 4, 5, 9, 15 and 31) loaded onto different factors across both datasets. It is important to notice that items 4 and 31 were not retained in the final AAQ version since they lowered CFA results in further international analyses.

The item reliability was analyzed through Cronbach's alpha coefficients for the three subscales suggested by the EFA. The Brazilian dataset showed coefficients of .863 for the Subscale I (and .845 for the International dataset), .804 for the Subscale II (.822 for the International sample) and .671 for the Subscale III (.701 for the International subscale).

The Item Total Correlation Analysis was then carried out in distinct steps. Firstly, the Brazilian dataset was analyzed to verify correlations below a critical cut-point (r = 0.40). Secondly, the International dataset underwent the same analysis. Thirdly, both findings were compared to verify potential discrepancies. Six items in the Brazilian dataset showed insufficient correlations (items 1,5,6,11,18 and 19). All these six items proved to show low coefficients in the International dataset too. Out of these, only item 18 remained in the final international AAQ version.

The Multi-trait Analysis Program (MAP) [27] was also used to assess scale fit and internal reliability of the three-factor model. Although six items loaded highly on other factors besides the predicted one (9, 13, 21, 24, 33 and 34, r ≥ .40 < .52), no items presented higher correlations with an unpredicted factor than with the predicted one. Furthermore, the directions presented by the MAP analysis (correlation coefficients) were in accordance with the EFA loadings.

### Confirmatory Factor Analysis

CFA was carried out using AMOS 6.0 software [28]. First, the 38 items three-correlated-factor solution was tested, showing insufficient results (χ^2 ^= 1516.60 p < .001, df = 662, CFI = 0.79, RMSEA = 0.05). In order to verify the impact of the correlation among factors, the uncorrelated solution was then tested, showing further decrease in model fit (χ^2 ^= 1943.63 p < .001, df = 665, CFI = 0.68, RMSEA 0.06).

Following the steps adopted by the international development of AAQ [14], the 31-item three-factor solution was then assessed in order to verify potential improvement in model fit. Similarly to the international findings, this structure showed insufficient improvement (χ^2 ^= 1005.62 p < .001, df = 431, CFI = 0.82, RMSEA = 0.05). Again, allowing interfactor correlation determines great model fit improvement.

**Figure 3:**
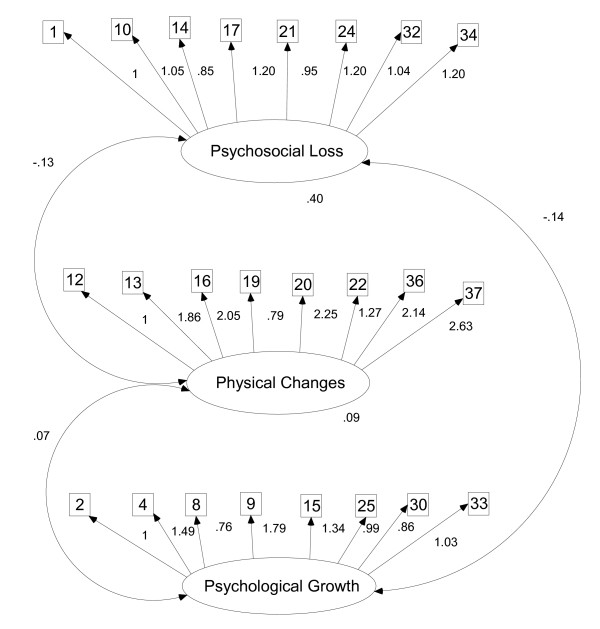
CFA model for the Brazilian sample (n = 424).

The final 24-item version was also tested in the Brazilian dataset, according to the structure illustrated in Figure 3.

Remarkable improvements in model fit were shown (χ^2 ^= *645.19 p = .061, df = 249, CFI = .83, RMSEA = .06*). The comparison of these indexes to the international ones indicate that the performance of the Brazilian final version is similar (international findings present *CFI = .84 and RMSEA = .05*)

### Discriminant validity

To assess the discriminant validity, a correlation between each domain score and the depression levels was performed. It was predicted that depression levels would be negatively correlated to the three factors, and that the physical factor should present a lower coefficient than the two psychological factors. In fact, the correlation results showed coefficients of r = -.59 with psychosocial loss, r = -.59 with psychological growth and r = -.35 with physical change.

### Item Response Theory

Responses were tested according to the Rasch model for polytomous scales [29]. Basically, the responses patterns observed in data collected are tested against an expected probabilistic form of the Guttman Scale [30]. Different fit statistics are applied to determine whether the observed data fits the expected model or not [31]. According to Rasch measurement theory, a scale should have the same performance, independently of the sample being assessed (e.g., age or gender) [20,21]. Reverse thresholds, an overall Chi-Square test (indicating whether the observed data differs from the expected model), item Chi-Square fit and Item fit-residuals were tested. In addition to these fit indexes, the item bias DIF (differential item functioning) was verified, since it can determine decrease in model fit, as well as measurement inappropriateness. The Person Separation Index (PSI) was calculated for each factor as an indicator of internal consistency reliability. In fact, the PSI gives information comparable to the Cronbach's Alpha from classic psychometric theory.

Table 4 presents the Rasch findings for the 24-item version in its original form. At this stage, the 5-point Likert response scale was maintained in its original form. As mentioned above, the Chi-Square (both for the model and for items separately) has the purpose of assessing whether the data collected fits the expected theoretical model. Thus, p values lower than 0.05 (corrected for Bonferroni Multiple Comparisons) indicate that the first is significantly different from the second, rejecting the desired similarity [32]. Item residuals (a sum of item and individual person deviations) also permit the assessment of item fit, and values from -2.5 to +2.5 show adequate fit.

**Table 4:** Rasch Analysis of the original 24-item final version including the 5-point Likert response scale

**Content**							**DIF Analyses**		
	**Item**	**Model χ^**2 **^Fit (df)**	**P value**	**Item χ^**2 **^Fit**	**Item Residual**	**Rev Threshold**	**Gender**	**Age**	**Depression**
**Subscale I**		**77.06 (40)**	**.00003**						
	7			3.08	1.01	✓			
	10			12.77	-0.06				
	14			15.27	**3.11**				
	17			5.52	-0.60				
PSI = .869	21			**21.12**	**3.49**				
	24			11.74	-1.25	✓			
	32			10.70	1.61				
	34			6.38	-1.07	✓			
**Subscale II**		**109.4 (48)**	**.00001**						
	12			10.57	-0.41	✓			Uniform
	13			6.57	.58	✓			
PSI = .807	16			4.65	.02	✓			
	19			**42.61**	**4.96**	✓			Uniform
	20			11.79	-1.04				
	22			17.47	**3.76**	✓			
	36			10.40	.66				
	37			5.34	.32	✓			
**Subscale III**		***59.06 (48)***	***.131***						
	2			1.94	.54	✓			
	4			10.11	-0.31	✓			
PSI = .745	8			3.17	1.24	✓			
	9			**19.17**	-2.05	✓			
	15			9.01	**3.43**				
	25			1.34	.37				
	30			6.73	1.58	✓			
	33			7.55	-1.73	✓			

In bold, item-residuals > 2.5 or item χ^2 ^fit with p < .05 corrected for Bonferroni Multiple Comparisons

Results described in Table 4 show that 6 items (9, 14, 15, 19, 21 and 22) presented high residuals and/or item χ^2 ^scores significantly different from the expected. The model fit for the three subscales also indicated misfitting. Furthermore, 15 out of 24 items presented threshold disorders, which suggests that the response scale is not adequate and therefore contribute to the misfittings found both in model and item levels.

Thus, rescoring items was carried out in order to improve the model. Firstly, the category probability curves were checked for each item. This approach allows the investigator to verify what response categories present disorders and, thus, what specific categories should be collapsed to improve the scale. Factors I and II demanded that categories two and three were merged, whereas factor III needed categories 3 and 4 collapsed together.

Analysis using the new 4-point scale showed that Factors I and III had remarkable improvement, with no model or item misfittings. On the other hand, Factor II presented a slight increased fit, but still insufficient (Model χ^2 ^= 87.12, DF 48, P = 0.0004, PSI = .752). The second step was then deleting the items responsible for the remaining misfitting, namely items 19 and 22. The final model, then, proved adequate fit. No reversed threshold or DIF remained after rescoring and item deletion (Factor II). Person Separation Indexes showed adequate scores for group comparisons (i.e., PSI > .70). Table 5 presents the indexes for the final model.

**Table 5:** Final 22-item version, including the rescored 4-point response scale

**Content**							DIF Analyses *		
	**Item**	**Model χ^**2 **^Fit (df)**	P value*	Item χ^**2 **^Fit*	Item Residual*	**Rev Threshold**	**Gender**	**Age**	**Depression**
**Subscale I**		**66.36 (40)**	**.006**						
	7			2.94	-0.276				
	10			9.33	-0.592				
	14			5.26	1.409				
	17			5.33	-1.734				
PSI = .815	21			17.10	2.359				
	24			12.57	-2.492				
	32			6.09	1.00				
	34			7.70	-1.507				
**Subscale II**		**65.56 (42)**	**.011**						
	12			4.01	0.434				
	13			3.44	0.7				
PSI = .750	16			3.51	1.239				
	20			9.20	-0.935				
	36			2.89	-0.439				
	37			9.07	-0.842				
**Subscale III**		**59.38 (48)**	**.125**						
	2			1.62	0.362				
	4			9.55	-0.534				
PSI = .710	8			10.84	0.783				
	9			16.29	-1.409				
	15			5.28	1.273				
	25			1.73	-0.242				
	30			6.88	1.175				
	33			7.16	-1.995				

* all p non-significant for 0.05 after Bonferroni correction

Local independence of items and unidimensionality (two Rasch assumptions) were assessed for the three final factors through two statistical tests. Item residuals correlations were firstly analysed to check the potential presence of local dependence (i.e., two items highly correlated in the final model, so that the response to one would be determined by the other). No correlations above 0.300 were found, which indicates local independence. Secondly, the pattern of residuals was analysed thorough PCA of the residuals. The first PCA factor was divided into two subsets (defining the most positive and negative loadings on the first residual component). These two subsets were then separately fitted into Rasch Model and the person estimates were obtained. An Independent T-test was then carried out to detect potential differences between the two subsets, which would indicate the presence of multidimensionality in the model [20]. No significant differences were found for the three factors of the scale (Factor 1, p = 0.051, Factor 2 p = 0.654, Factor 3 p = 0.090).

## Discussion

The present paper had two complementary aims. First, it had the goal of presenting a validated Brazilian version of the Attitudes to Aging Scale. This version will permit that aging experiences may be assessed in a distinct and poorly investigated population. Furthermore, since aging is a widespread phenomenon and is highly dependent on socio-cultural aspects, it is extremely important that new measures of this construct can be successfully applied in different contexts. This would permit that adequate cross-cultural investigations on attitudes to aging may be carried out, including a valid and reliable instrument.

Secondly, this article aims to present a comprehensive approach in validating new measures, which include both classical psychometric theory and modern methodologies together in a complementary way. While the traditional approach provides relevant information regarding discriminant validity, missing values distributions and factor analyses loading, Rasch analysis represents a powerful tool in assessing item bias, threshold disorders and model fit [20].

The Attitudes to Aging Questionnaire is a unique measure of perception regarding aging, since it was developed through a well-established international methodology and based since its principle in focus groups run with older adults [15-17,33]. Furthermore, it relies on the assumption that the subjective perception of the aging process is the ultimate construct to be measured, other than objective indicators of physical activity or psychological distress.

Regarding the psychometric performance, the Brazilian version demonstrates good performance on both classical and Rasch approaches. Despite the insufficient goodness-of-fit indexes in CFA (CFI < .90), suitable discriminant validity, and excellent fit indicators from Rasch analysis suggested that the Brazilian version has satisfactory performance and, thus, can be applied in different studies reliably.

Another relevant issue regarding the findings of the AAQ validation is the construct similarity between the international sample and the Brazilian one. The three factors proposed by the international analysis seem to be replicated in the Brazilian dataset. Indeed, Psychosocial Loss, Physical Change and Psychological Growth represented the theoretical ground upon which items were grouped during the factor analysis phase. It could indicate that the perception of aging did not differ significantly between the two samples and raises the question of whether these similarities remain or not in other different cultures. The demonstration of cultural invariance of the core attitudes to aging could lead to the possibility of reliable comparisons, which is needed by both researchers and policy makers.

It is suggested, however, that rescoring and two item deletions could increase Brazilian scale fit and performance. These potential alterations should not promote crucial modifications in the scale format, since they can be made during the statistical analysis phase and not necessarily in the data collection stage. Since this is the first psychometric analysis of the Brazilian AAQ version, authors encourage the scale users to verify whether the 22-item version maintains its superiority over the original 24-item format in distinct samples, and then explicitly decide for one format.

## Conclusion

The described findings support the hypothesis that the development of a new international instrument according to a simultaneous methodology, which includes an intense qualitative initial phase, is adequate to generate reliable cross-cultural measures. In conclusion, the Brazilian version of the AAQ instrument is a reliable, valid and consistent tool to assess attitudes to aging and can be applied in international cross-cultural investigations running less risk of cultural bias.

## Competing interests

The author(s) declare that they have no competing interests.

## Authors' contributions

EC participated in the study design, data collection, statistical analysis and drafted the manuscript; MPF participated in the study design, statistical analysis and helped to draft the manuscript; CMT participated in the study design and data collection; KL helped to draft the manuscript and took part in the theoretical discussion; MJP participated in the study design, statistical analysis and helped to draft the manuscript. All authors read and approved the final manuscript.
